# Absence of the Z-disc protein α-actinin-3 impairs the mechanical stability of *Actn3KO* mouse fast-twitch muscle fibres without altering their contractile properties or twitch kinetics

**DOI:** 10.1186/s13395-022-00295-8

**Published:** 2022-06-23

**Authors:** Michael Haug, Barbara Reischl, Stefanie Nübler, Leonit Kiriaev, Davi A. G. Mázala, Peter J. Houweling, Kathryn N. North, Oliver Friedrich, Stewart I. Head

**Affiliations:** 1 Institute of Medical Biotechnology, Friedrich-Alexander-University Erlangen-Nürnberg, Erlangen, Germany; 2 School of Medicine, Western Sydney University, Sydney, NSW 2560 Australia; 3 School of Medical Science, University of New South Wales, Sydney, NSW Australia; 4 Department of Kinesiology, College of Health Professions, Towson University, Towson, MD USA; 5 Murdoch Children’s Research Institute, Melbourne, VIC Australia; 6 Department of Paediatrics, University of Melbourne, Melbourne, VIC Australia

**Keywords:** a-Actinin-3, Exercise, Sarcoplasmic reticulum, Skeletal muscle, Skinned fibre, Biomechatronics, Biosensors, Single fibre, Myorobotics

## Abstract

**Background:**

A common polymorphism (R577X) in the *ACTN3* gene results in the complete absence of the Z-disc protein α-actinin-3 from fast-twitch muscle fibres in ~ 16% of the world’s population. This single gene polymorphism has been subject to strong positive selection pressure during recent human evolution. Previously, using an *Actn3KO* mouse model, we have shown in fast-twitch muscles, eccentric contractions at *L*_0_ + 20% stretch did not cause eccentric damage. In contrast, *L*_0_ + 30% stretch produced a significant ~ 40% deficit in maximum force; here, we use isolated single fast-twitch skeletal muscle fibres from the *Actn3KO* mouse to investigate the mechanism underlying this.

**Methods:**

Single fast-twitch fibres are separated from the intact muscle by a collagenase digest procedure. We use label-free *second harmonic generation* (SHG) imaging, ultra-fast video microscopy and skinned fibre measurements from our *MyoRobot* automated biomechatronics system to study the morphology, visco-elasticity, force production and mechanical strength of single fibres from the *Actn3KO* mouse. Data are presented as means ± SD and tested for significance using ANOVA.

**Results:**

We show that the absence of α-actinin-3 does not affect the visco-elastic properties or myofibrillar force production. Eccentric contractions demonstrated that chemically skinned *Actn3KO* fibres are mechanically weaker being prone to breakage when eccentrically stretched. Furthermore, SHG images reveal disruptions in the myofibrillar alignment of *Actn3KO* fast-twitch fibres with an increase in Y-shaped myofibrillar branching.

**Conclusions:**

The absence of α-actinin-3 from the Z-disc in fast-twitch fibres disrupts the organisation of the myofibrillar proteins, leading to structural weakness. This provides a mechanistic explanation for our earlier findings that in vitro intact *Actn3KO* fast-twitch muscles are significantly damaged by *L*_0_ + 30%, but not *L*_0_ + 20%, eccentric contraction strains. Our study also provides a possible mechanistic explanation as to why α-actinin-3-deficient humans have been reported to have a faster decline in muscle function with increasing age, that is, as sarcopenia reduces muscle mass and force output, the eccentric stress on the remaining functional α-actinin-3 deficient fibres will be increased, resulting in fibre breakages.

**Supplementary Information:**

The online version contains supplementary material available at 10.1186/s13395-022-00295-8.

## Background

Around 16% of humans lack α-actinin-3, due to a homozygosity for a common polymorphism in the *ACTN3* gene. This single gene polymorphism has been subject to strong positive selection during the last 50,000–60,000 years corresponding to the migration of modern humans from the African continent [1, 2]. Intriguingly, two recent publications suggest that a major positive selection pressure may have been the fact that the α-actinin-3 polymorphism improves an individual’s cold acclimatisation [3, 4]. The *ACTN3* gene has become known colloquially as the “gene for speed” [1, 5]. α-Actinin-3 deficiency is not associated with any skeletal muscle pathology; indeed, it appears to be beneficial for female elite endurance athletes [6]; however, it should be noted several subsequent studies in humans (which may be underpowered due to the large genetic variability) have not supported this first report [7]. The speed of shortening of a muscle fibre depends largely on the myosin heavy chain (MyHC) isoform present [8–11]. In previous studies, we have shown that MyHC expression is unaltered in *Actn3 knockout (KO)* fibres [12] and, by using a skinned fibre preparation, demonstrated that there is no difference between *Actn3KO* and wild type (WT) fast-twitch fibres regarding the Ca^2+^ sensitivity of the contractile proteins [13]. In an intact preparation, using a high-speed imaging technique [8] and enzymatically isolated single fibres from *Actn3KO* and WT mice, we showed no difference in the maximum speed of unloaded shortening during a single action potential triggered twitch [3]. Taken as a whole, these data suggest the *ACTN3KO* gene does not alter the myosin isoform or contractile functioning of the contractile proteins.

The α-actinins are rod-shaped proteins of 35 nm length that form antiparallel homodimers. Mammalian skeletal muscle expresses α-actinin-2 and α-actinin-3. These isoforms are the major component of the Z-disc. α-Actinin-2 comprises the Z-discs of slow-twitch muscles while α-actinin-3 is found exclusively in the Z-discs of fast-twitch muscles [14]. The Z-discs play a key role in longitudinal force transmission from the sarcomeres to the tendons [5]. In human fast-twitch muscles, α-actinin-3 is more abundant in type 2X fibres compared to type 2A [15]. Fast-twitch fibres are highly susceptible to damage from eccentric contractions while slow-twitch fibres are very resistant to any damage from eccentric contractions [16, 17]. While it is not clear why fast-twitch fibres are more susceptible to damage due to eccentric contractions compared to slow-twitch fibres, one structural reason comes from the observations that fast-twitch fibres have narrower Z-discs which would provide less mechanical support, i.e. higher mechanical stress, during high tension contractions [14]. The width of the Z-disc largely reflects the amounts of α-actinin proteins anchoring the actin filaments of adjacent sarcomeres at the Z-discs. In *ACTN3KO* muscles of XX individuals, there is a complete absence of α-actinin-3 which is functionally compensated for by the closely related protein α-actinin-2 [1]. If α-actinin-2 configures in the narrow Z-disc fast-twitch profile in XX individuals, then these Z-discs may be less stable than the “wild-type” narrow α-actinin-3 fast-twitch Z-discs present in RR individuals homozygous for the *ACTN3* gene. RR humans can be thought of as wildtype, and they express the full complement of the protein alpha-actinin-3 in fast-twitch skeletal muscle fibres and are equivalent to our homozygous wildtype mice. In our earlier studies [18, 19] on isolated intact fast-twitch extensor digitorum longus (EDL) muscles from our mouse *Actn3KO* model, we compared the effect of eccentric contractions at *L*_0_ + 20% and *L*_0_ + 30% stretch. Eccentric contractions at *L*_0_ + 20% stretch did not result in a significant eccentric damage force deficit; in contrast, *L*_0_ + 30% stretch did produce a significant ~ 40% force deficit. We interpreted these results as suggestive that an absence of α-actinin-3 increases the susceptibility to damage when *Actn3KO* mouse fast-twitch muscles are subject to high forces. However, in intact muscles, there is an interference from intermuscular pathways of lateral force transmission via the dystrophin and desmin pathways as well as the mechanical role played by the connective tissue lattice supporting muscle fibres within the intact muscle. In the current study, we used single permeabilised fibres to directly probe the effects of the absence of α-actinin-3 on the longitudinal mechanical strength and contractility of fast-twitch fibres.

## Methods

### Animals

The *Actn3*KO mouse line was previously created in this laboratory [20], and experiments were performed on male animals at 12–15 months of age. A total of 16KO and 16WT mice were used in the present study. The use of animals was approved by the Animal Care and Ethics Committees of the Children’s Medical Research Institute and the University of New South Wales.

### Skeletal muscle single fibre enzymatic isolation

Flexor digitorum brevis (FDB) and extensor digitorum longus (EDL) muscles were digested in Krebs solution composed of (in mM): 4.75 KCl, 118 NaCl, 1.18 KH_2_PO_4_, 1.18 MgSO_4_, 24.8 NaHCO_3_, 2.5 CaCl_2_ and 10 glucose containing 3 mg/ml collagenase type IV A (Sigma Aldrich, USA), gently bubbled with carbogen (95% O_2_, 5% CO_2_) and maintained at 37 °C. After 25–30 min, muscles were removed from the digest solution with a wide bore glass pipette and serially rinsed twice in Krebs solution containing 0.1% foetal calf serum. Single fibres were dispersed by gentle trituration. The FDB fibres were maintained in Krebs solution with 0.1% foetal calf solution at room temperature 21–23 °C and continuously bubbled with carbogen. Using a pipette, 0.5 ml of solution was drawn and placed on a cleaned glass slide on an inverted microscope, and each 0.5 ml contained between 10 and 50 fibres. FDB fibres attached firmly to the glass cover slip and were continually superfused with Krebs bubbled with carbogen at a rate of around 0.5 ml per minute. The FDB fibres were visualised at 200 × magnification on a Nikon Eclipse Ti2-E Inverted Research Microscope. For fibre length and diameter measurements (Supplementary Fig. A), a grid was placed in the eye piece of the microscope so that it occupied ~ 50% of the field of view and all fibres in this view were recorded and processed using ImageJ open-source software, the microscope was calibrated using a stage micrometre, and a total of 200 WT FDB fibres were measured. Post-digest EDL muscles were rinsed first in Krebs with 0.1% foetal calf serum to stop the collagenase reaction and then rinsed for a second time in Krebs with no foetal calf serum and no added calcium before being placed in a relaxing solution with the following composition (mM): 117 K^+^, 36 Na^+^, 1 Mg^2+^, 60 HEPES, 8 ATP, 50 EGTA (Note: as the fibres are effectively chemically skinned by the high EGTA concentration, this is an intracellular solution). Transfers between solutions were made by sucking the digested muscle mass into a wide bored pipette. Finally, the muscle was gently agitated using a wide bore pipette to release individual fibres from the muscle. Fibres were maintained in the relaxing solution at 4 °C for up to 4 h before use.

### High-speed acquisition of transillumination images

We selected FDB fibres with a width of 35 μm or greater (Supplementary Fig. A), FDB is a fast-twitch muscle, and we only used fibres which responded briskly and repeatedly to a 1-ms activating pulse, over 90% of FDB fibres are fast-twitch; however, we occasionally came across fibres which were slower to contract and relax (visual inspection), these fibres were not used [21]. Intact single FDB fibres were electrically field-stimulated with supramaximal voltage pulses of 1 ms duration and 10 V amplitude over a range of frequencies from 10 to 100 Hz. The duration of the stimulus was 250 ms. The stimulator probe was bipolar, with two fine platinum wires isolated up to the ends, the wires were attached to a fine Perspex rod mounted on a micromanipulator to enable it to be placed close (~ 10 µm) to the neuromuscular junction of the selected FDB fibre. A CMOS PCO1200hs high-speed camera (PCO AG, Kehlheim, Germany) was mounted to the camera side-port of the Nikon inverted microscope. The Peltier-cooled camera was connected to a computer for acquisition control and data storage. Single fibres approximately covered a 520 × 160 pixel area when visualised through a 20 × objective which allowed frame rates for shortening sequences of 4200 frames per second. Recordings were synchronised with the induction of a single twitch and image read-out and storage from the ring-buffer of the camera was performed offline. For offline analysis of each experiment, an image sequence of approximately 1000 to 1700 frames per fibre was analysed using a modification of a previously written processing algorithm in an interactive data language environment [8].

### EDL skinned fibre solutions

A single large (top 30% diameter of the fibres) intact EDL fibre was selected from the population of fibres using a fine bore pipette. We have previously shown that in mice there is a strong correlation between fibre size and type with fast fibres having nearly twice the cross-sectional area (CSA) compared to slow-twitch type 1 [12]. The selected fibre was tied onto a sensitive force transducer of the *MyoRobot* biomechatronics system [22] or in-house skinned fibre rig. After tying, it was placed for 10 min in solution A (see later) with 2% Triton X-100 added to chemically skin all remaining membranous cell elements. The fibre was then exposed to a series of solutions of different free Ca^2+^ concentrations. The strongly buffered Ca^2+^ solutions were prepared by mixing specific proportions of EGTA-containing solution (solution A) and Ca-EGTA–containing solution (solution B). Solution A contained 117 mM K^+^, 36 mM Na^+^, 8 mM adenosine triphosphate (ATP, total), 1 mM free Mg^2+^, 10 mM creatine phosphate, 50 mM EGTA (total), 60 mM N-[2-hydroxyethyl] piperazine-N′-[2-ethanesulfonic acid] (HEPES) and 1 mM NaN_3_ (pH 7.10). Solution B was similar to solution A, with the exception that the EGTA and Ca^2+^-EGTA concentrations of solution B were 0.3 and 49.7 mM, respectively. The free Ca^2+^ concentrations of the solutions were calculated using a *K*_apparent_ for EGTA of 4.78 × 10^6^ M^−1^. The maximal force was determined by exposure to solution B, containing a free Ca^2+^ concentration of 3.5 × 10^−5^ M. Force was returned to baseline after maximal activation by exposure to solution A. The plateaus of the force responses elicited by exposure to solutions of increasing free Ca^2+^ concentration are expressed as a percentage of maximum Ca^2+^-activated force and plotted as a function of pCa. The force–pCa data were fitted with Hill curves using GraphPad Prism8.

**Fig. 1:**
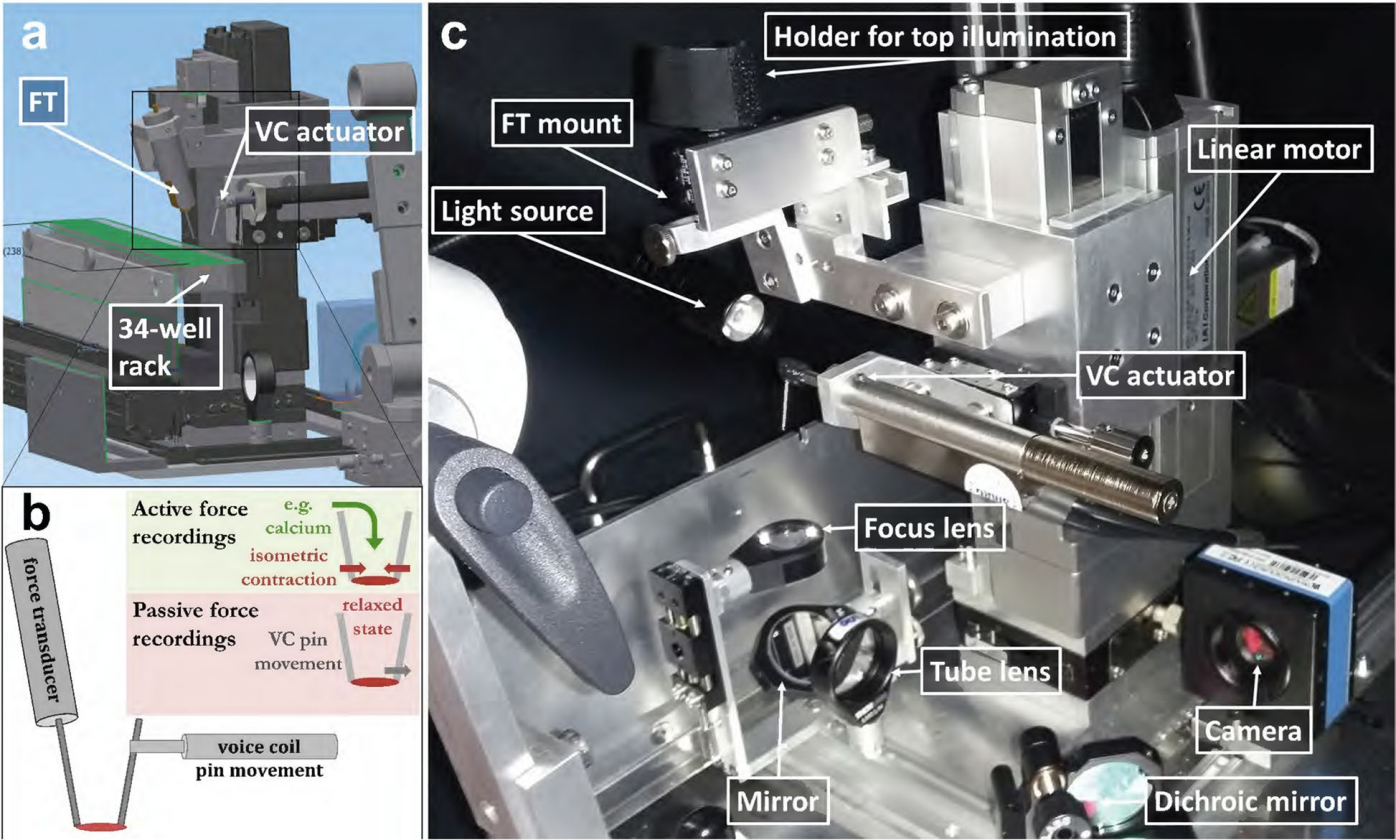
*MyoRobot* biomechatronics system.** a** Displays a schematics setup of the automated robotised system, whose functionality is centred around an optical force transducer, a voice-coil actuator and a 34-well rack housing bioactive solutions. **b** Diagram of the attachment points of force transducer and voice-coil between which the single muscle fibre is mounted; the fibre is shown in red. Upon immersion in different wells or by precisely elongating its extension, active and passive force recordings can be carried out in an automated fashion. **c** Shows a photograph of the *MyoRobot* with the 34-well rack removed to show the optics system used to record the muscle fibre diameter in a transmission illumination setting

The MyoRobot, automated biomechatronics system, is shown in Fig. 1. For full details of the *MyoRobot*, see Haug and Reischl [22]. The following procedures were carried out on the EDL fibres using the *MyoRobot*: (i) *Force-pCa*, the fibre was immersed in wells containing highly EGTA-buffered internal solutions with decreasing pCa values, made up by mixing solutions A and B (see above), and exposure to each pCa was for 20 s; *and (ii) passive axial elasticity, resting length-tension curves*, to assess axial fibre compliance through resting length-tension curves when the fibre was relaxed in low Ca^2+^ the voice coil was driven at very slow speed (quasi-static) to stretch the fibre while passive restoration force was sampled at 200 Hz. Since the skinned fibres possess viscous properties (e.g. presence of titin), the stretch velocity was optimised to values slow enough to be in a steady state between instantaneous elastic restoration force and viscous relaxation.

### Eccentric contractions

The selected fibre was tied to a sensitive force transducer on our custom-designed in-house skinned fibre rig. The fibre was placed in a maximal Ca^2+^ activating solution and allowed to produce maximal isometric force; it was then stretched by 20% of *L*_0_ for 2 s, and the stretch was released for a further 2 s before the fibre was relaxed in a low Ca^2+^, high EGTA solution. The velocity of stretch was 1.2 mm/s. The procedure was carried out three times in total, and a final maximal Ca^2+^-activating force was recorded.

### Second harmonic generation imaging of single fibres

Single EDL fibres were tied to thin glass rods and fixed in 0.1% glutaraldehyde solution for SHG microscopy. Glass rods with one EDL fibre each were mounted into a microscopy chamber immobilised between Vaseline® stripes for multiphoton imaging; for details, see Friedrich et al. [23].

### Statistics

Data were presented as means ± SD. Differences occurring between genotypes were assessed by one-way ANOVA with respect to genotype. Post hoc analysis was performed using Holm-Sidak’s multiple comparisons test. The Logrank test was used to compare survival distributions of muscle fibres during contraction and the Mann–Whitney test was used for comparing angular variability of myofibres between groups. All tests were conducted at a significance level of 5%. All statistical tests and curve fitting were performed using a statistical software package Prism version 8 (GraphPad, USA).

## Results

**Fig. 2:**
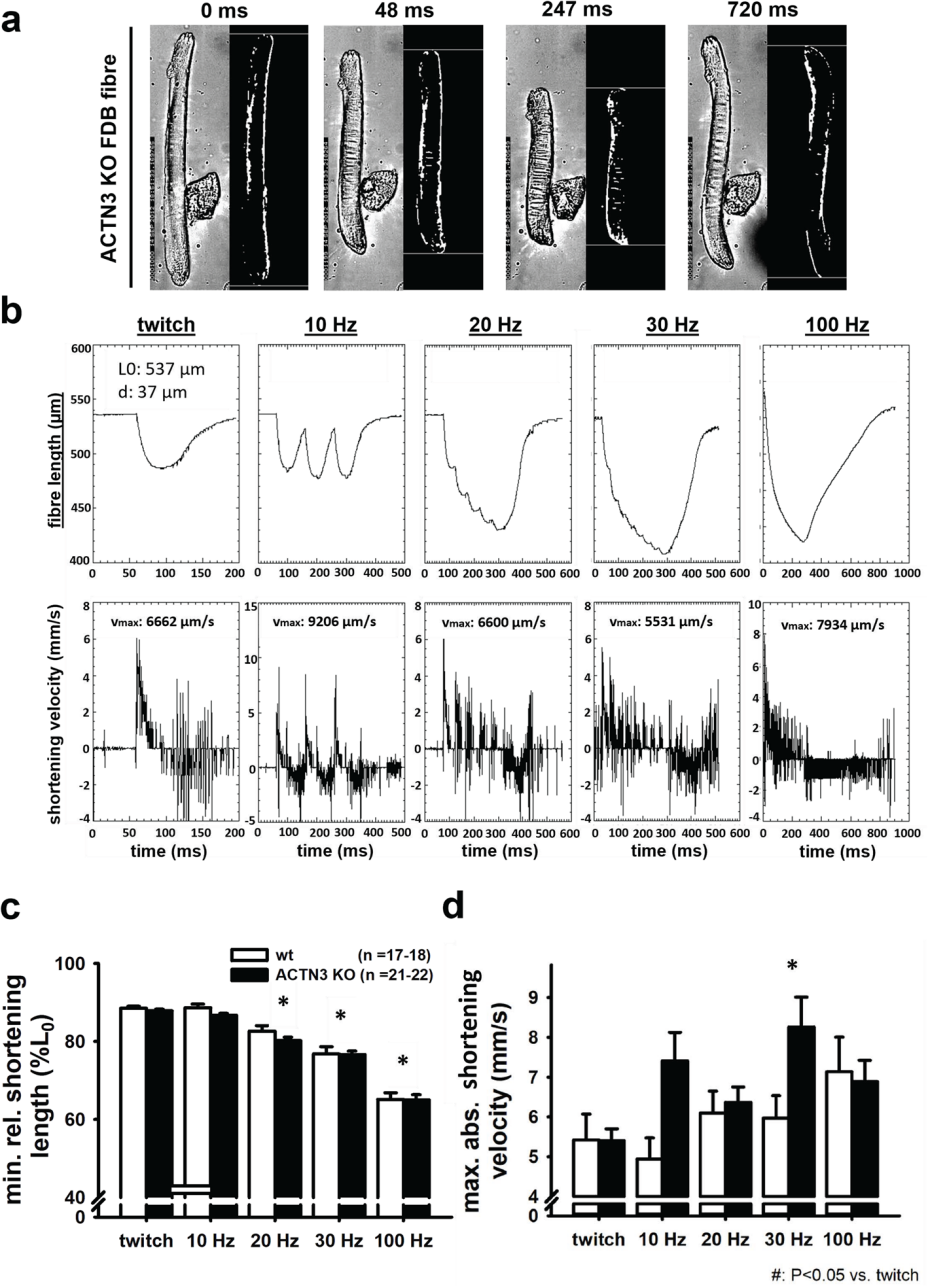
Unloaded speed of shortening during external field stimulation of single enzymatically dissociated intact FDB fibres. **a** Example images during a shortening sequence of a *Actn3KO* single fibre stimulated at 100 Hz for 250 ms and recorded at 4166 fps. Also shown (right panel, fibre image on a black background) are the automated analysis images from which the shortening parameters, fibre length and shortening velocity were recorded. **b** Analysed time traces of these parameters for the same fibre at indicated stimulation frequencies. **c** Minimum shortening length was not different between WT and Actn3KO mice; however, there was a significant reduction in both genotypes at higher frequencies 20–100 Hz as indicated *. **d** Absolute maximum shortening velocities were not different between genotypes apart from 30 Hz where *Actn3KO* were significantly faster as indicated by *. Significance WT vs. KO based on one-way ANOVA test indicated as follows: *p < 0.05

**Fig. 3:**
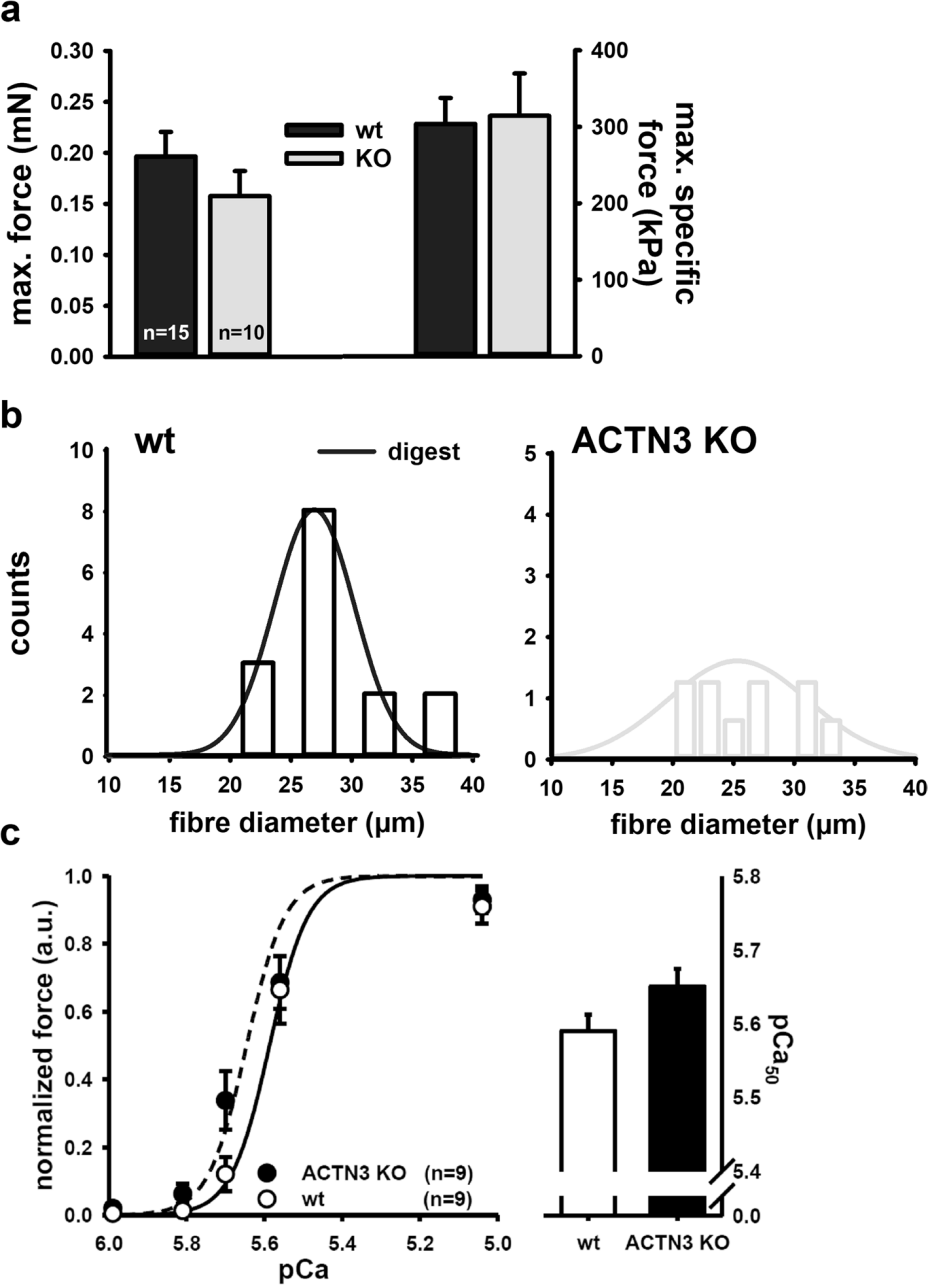
Maximum myofibrillar force in single EDL fibres from Actn3KO mice is unaltered compared to WT. **a** Statistical analysis of maximum force and specific force (normalised to fibre diameter-derived cross-sectional area) values from EDL muscles of WT and Actn3KO mice. No significant differences based on one-way ANOVA tests were apparent. **b** Analysis of fibre diameter distributions shows a trend to smaller diameter in the Actn3KO fibres. **c** Calcium sensitivity shown as pCa-force relationship. Average data are displayed along with the reconstructed average fit

**Fig. 4:**
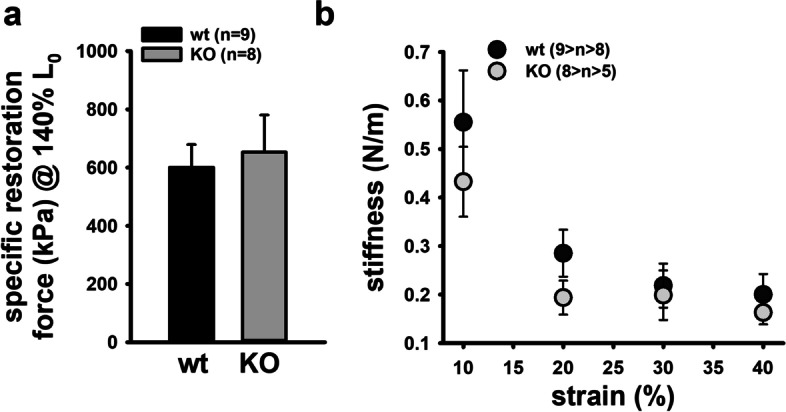
Resting length-tension curves and steady-state compliance of EDL Actn3KO and wildtype single fibres.** a** Specific restoration force (stress) at 140% *L*_0_ analysed in WT and Actn3KO fibres. **b** Steady-state stiffness values vs. strain indicate a slight trend to lower mechanical stiffness in the Actn3KO background compared to the WT

**Fig. 5:**
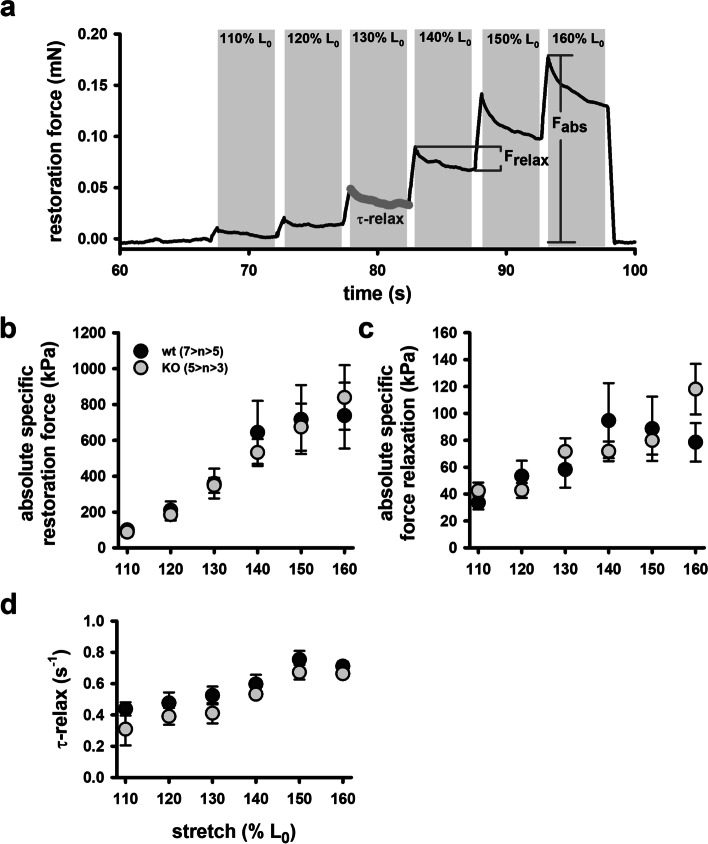
Single fibre visco-elasticity in EDL muscle from adult Actn3KO mice.** a** Example trace of ‘strain-jumps’ of increasing percentage of *L*_0_ amplitudes quickly applied to the fibre. **b** Each sudden stretch is answered by an instantaneous increase in restoration force F_R_ to a new maximum *F*_abs_ (**c**) before exponentially relaxing to achieve a new steady-state level with relaxation amplitude *F*_relax_ (**d**) and a time constant **t**_relax_

**Fig. 6:**
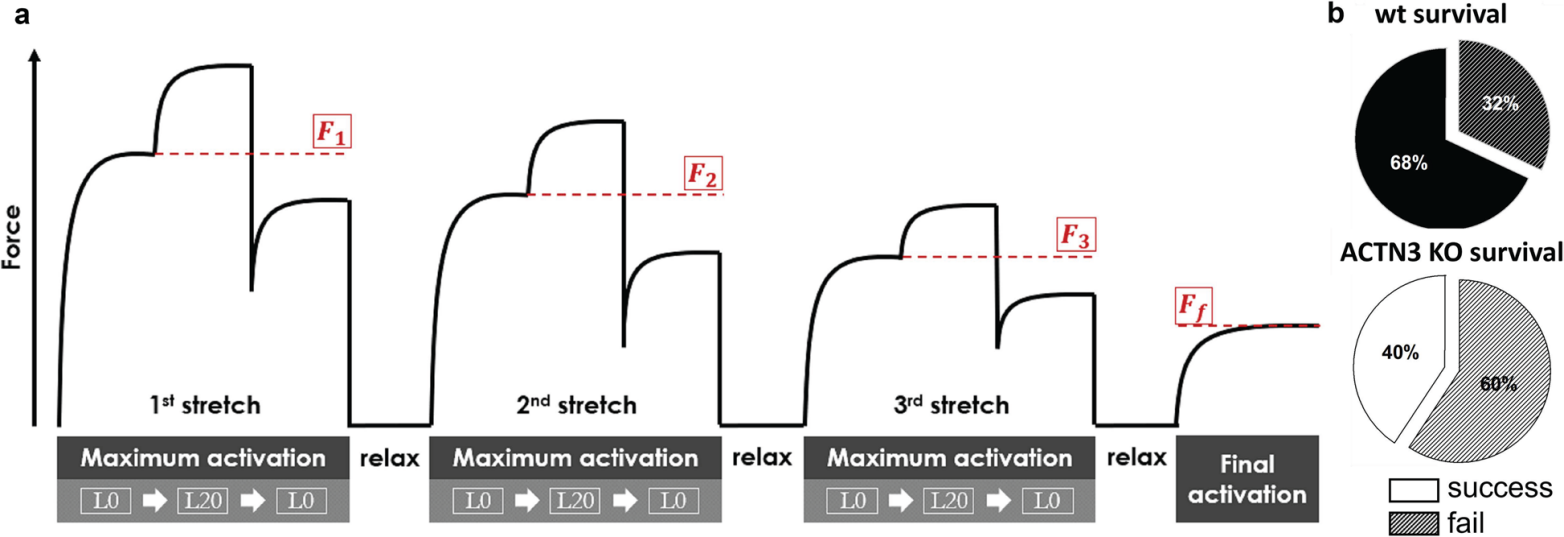
Contraction-induced breakages are more pronounced in EDL fibres from Actn3KO mice. **a** Schematic of the eccentric contractions protocol, maximally activating a single fibre and then imposing a 20% stretch before returning to resting length *L*_0_ and subsequent relaxation. Three eccentric contractions were carried out followed by a final assessment of maximum isometric force. **b** Actn3KO fibres showed much lower survival and higher rate of breakage during the sequence to a significance of *p* = 0.02 based on logrank analysis. In **b**, fibres that broke are shown in the segments of the pie chart with diagonal lines, while nonbreaking fibres are shown in solid fill

**Fig. 7:**
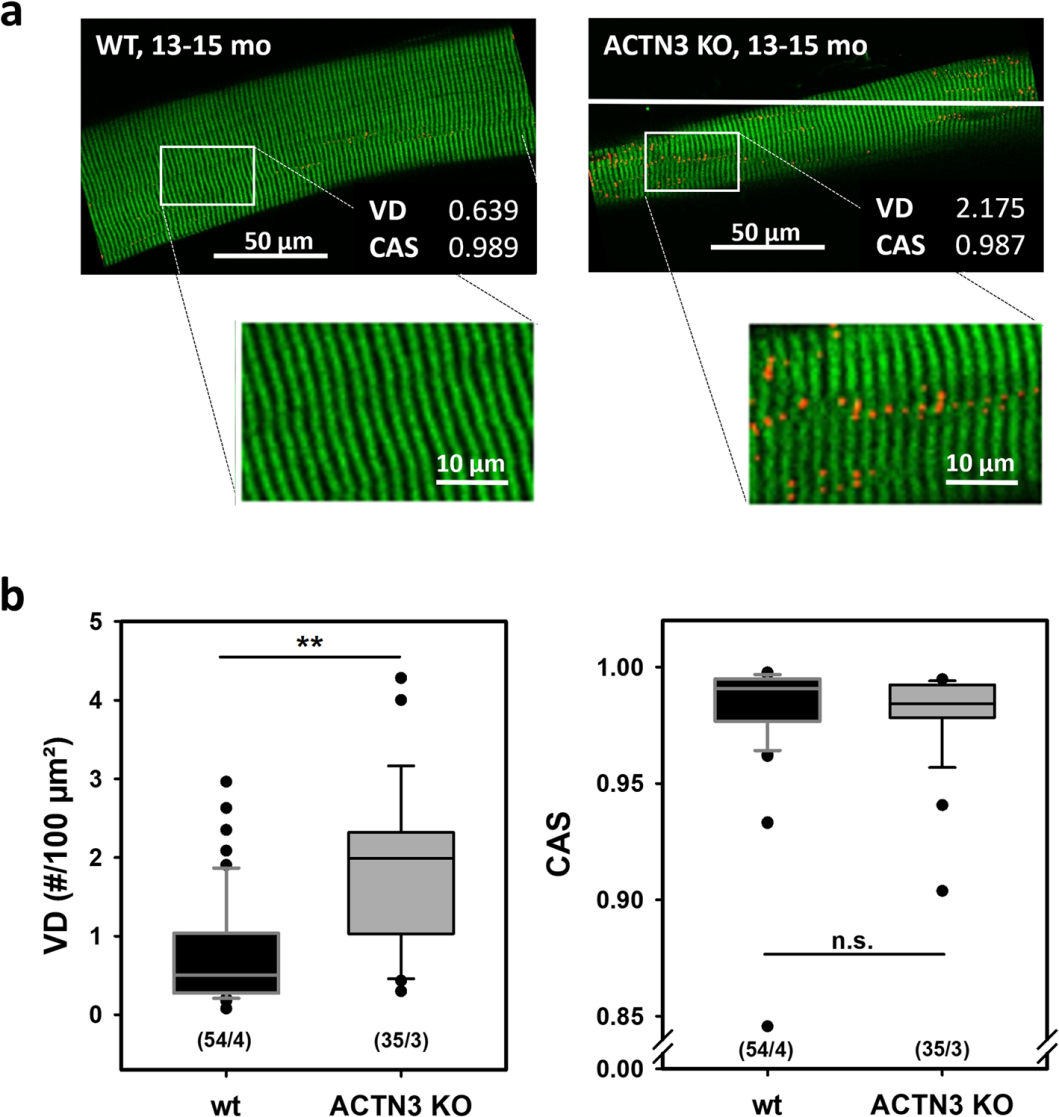
Second harmonic imaging and quantitative morphometry imaging in single dissociated EDL fibres reveal misregistered myofibrillar ultrastructure in Actn3KO. **a** Representative example images from the middle plane of a single WT and Actn3KO EDL fibre (top) with the automatically detected verniers shown as red dots and short red lines the VD and CAS values derived from the morphometry analysis are given in text on the bottom right of each panel. A magnified rectangular section is given below the images to enable a clearer view of the automatically detected (in red) myofibrillar disruptions in the Actn3Ko compared with the absence of such deviations in the WT fibre. **b** Group analysis in a substantial number of single fibres from several animals reveals significantly higher VD values in Actn3KO fibres over fibres from WT littermates, indicative of a higher linear *out-of-register* disorder. As for the angular variability of myofibrils, the CAS values were similar in both groups. **p < 0.001, Mann–Whitney rank sum test. (*n*/*m*): *n* single fibres from *m* animals

FDB muscles were dispersed into intact single fibres by collagenase digestion. A typical digest normally yields over 200 viable fibres. Supplementary Fig. A shows the range of muscle fibre widths obtained from a digest from a control FDB muscle. Since we first described using Bekoff and Betz [24] digest technique on mouse fibres in 1990 [25], it has proved a robust tool to generate intact isolated mouse muscle fibres for the study of their cell physiology [26, 27]. A viewing of the video from our high-speed camera of a single unloaded FDB fibre contracting at 20 Hz shows the reliability of this preparation in being able to produce repetitive unloaded contraction and relaxation cycles (Supplementary Fig. B (video)). Figure 2a, b shows single FDB fibres from *Actn3KO* and WT being stimulated at 10–100 Hz and their associated fibre length and shortening velocity. The combined data is shown in Fig. 2c and d. The shortening length and maximum velocity of shortening were not significantly different between *Actn3KO* and WT; however, at higher frequencies of stimulation (20–100 Hz), there was a significant slowing of the minimum relative shortening length, which was similar for both genotypes Fig. 1c. At 30 Hz, the absolute maximum velocity was significantly faster in *Actn3KO*; this difference was no longer present at 100 Hz (Fig. 2d). The values we measured for velocity of shortening were like those previously reported for mouse fast-twitch fibres [8]. For the skinned fibre experiments, we used the EDL fast-twitch muscle with longer fibres suitable for tying to a sensitive force transducer (not feasible for the ~ 500 µm long FDB fibres). The mouse EDL has been shown to have a fibre type distribution that is ~ 79% type 2B (fast glycolytic), ~ 16% type 2X and ~ 4% type 2A (fast oxidative glycolytic) muscle fibres [28]. Figure 3 shows the maximal force produced by isolated myofibres from *Actn3KO* and WT mouse EDL. Figure 3a shows that *Actn3KO* fibres tended to produce less absolute force than WT, but when corrected for CSA (specific force) the maximal force output of the myofibrillar proteins was the same for both genotypes, confirming our results from the intact whole EDL [18]. Figure 3b shows the fibre diameter distributions of WT and *Actn3KO*; here, we see a trend for the fibres to be of smaller diameter in *Actn3KO* as we have previously reported [12]; however, here these are not random samples, as for both WT and *Actn3KO*, we actively selected the longest fibres with the largest diameters for attaching to the force transducer. Figure 3c shows the combined pCa-force curves generated for the WT and *Actn3KO* fibres; there were no meaningful differences in the contractile properties, i.e. the slope of the pCa-force curves or pCa_50_ and these parameters were in the range of those previously reported for fast-twitch fibres [29]. We demonstrate that resting length-tension curves and steady-state compliance are not different between *Actn3KO* and WT myofibres, showing that the absence of α-actinin-3 from the Z-discs did not alter the elastic properties of the myofibrils (Fig. 4). We next looked at the single fibre visco-elasticity in *Actn3KO* and WT by rapidly stretching the single fibre in a series of 10% steps up to 60% longer than its starting value of *L*_0_ (100%) (Fig. 5). Here, a peak restoration tension was rapidly reached with each step followed by an exponential force relaxation as shown in Fig. 5a. Three parameters: absolute specific restoration force, absolute specific relaxation force and the rate of relaxation, are shown on the raw data trace in Fig. 5a. Total maximal specific force produced by each stretch was not different between *Actn3KO* and WT (Fig. 5b). After attaining maximum force at each length, the fibre was allowed to relax to a new steady state over 4 s. Then, we evaluated the amount of force drop during the relaxation period, which was not significantly different between WT and *Actn3KO* (Fig. 5c). Figure 5d shows the time constant of the rate of relaxation, and once again, there was no difference between WT and *Actn3KO*. To investigate the mechanical stability of the *Actn3KO* fibres, a set of three eccentric contractions were performed at + 20% of *L*_0_ (Fig. 6a), the fibre was first maximally activated by exposing it to a high Ca^2+^ solution. Once the force had plateaued, it was stretched by 20% of *L*_0_, held for 2 s and then released. The fibre was then allowed to reach a new maximal plateau for 2 s before being relaxed in a high EGTA relaxing solution. Figure 6b shows that during the three contraction eccentric protocol, there was a significant number of fibres which broke apart so that the fibre separated into two distinct pieces, *Actn3*. When we quantified these breakages, it was clear *Actn3KO* fibres broke more frequently (60%) because of the eccentric contraction protocol than WT (32%) (Fig. 6b). To investigate if there was a morphological reason for the increased breakage in the *Actn3KO* fibres, we used *Second Harmonic Generation imaging* (SHG) and quantitative morphometry in single EDL muscle fibres (Fig. 7). Group analysis of 54 WT fibres and 35 *Actn3KO* fibres showed that the *Actn3KO* fibres had significantly higher levels of myofibrillar axial lattice disorder, which we term vernier density (VD) likely due to Z-disc anchorage inhomogeneities resulting from the absence of α-actinin-3 (Fig. 7a). These myofibrillar Y-shaped vernier deviations or disruptions (Fig. 7a and b) will be points susceptible to mechanical weakness in the contractile filaments present in fibres.

## Discussion

Force transmission from the myosin heads to the Z-discs (a major component of which is the protein of α-actinin-2 and α-actinin-3) is mediated by actin filaments and titin [30]. The Z-discs are the focal point of force transmission and mechanical strength within the fibre [5]. When muscles are damaged by excessive forces, such as those experienced during an eccentric contraction, electron micrographs show that the initial point of damage is at the Z-disc [31]. In relation to the eccentrically stretched fibres which broke in the present study, we were unable to ascertain where (at the attachments or in the central portion of the fibre) the yield point occurred. This was due to the super-contraction of the unsupported broken fibre segments in the maximal activating Ca^2+^ solution. Fast-twitch muscles express their own isoform of α-actinin, α-actinin-3. Globally, ~ 1.6 billion people have a polymorphism (R577X) in the *ACTN3* gene which means they cannot produce the protein α-actinin-3 in their fast-twitch muscles [2]; in these people, α-actinin-2 is upregulated to (partially) compensate for the loss. We have generated an *Actn3KO* mouse model to study the morphological and contractile consequences of the absence of α-actinin-3 from fast-twitch muscles. However, it should be born in mind that the Z-disc also has a key role as a force sensor linking tension along the myofibrils with intracellular chemical signalling mechanisms [32]. Our FDB digest technique has been refined from our first digest of mouse skeletal muscle fibres in 1990 [25]. In its current form, the digest produces over 200 single contracting fibres per batch, the majority of which could undergo at least three consecutive rounds of fatiguing contractions followed by recovery. High magnification high-speed video framing (up to 4166 frames per second) showed that over 70% of the single FDB fibres attached to cleaned glass cover slips at their centre portions. They shortened and relaxed linearly about this point (Fig. 1a, b and Supplementary Fig. B (video)) when stimulated with a supramaximal voltage pulse 1 ms in duration delivered from a bipolar pair of platinum wires insulated to the tips and positioned close to the neuromuscular junction. A portion of the fibres would bend into crescent shapes during repeated contractions, and those were discarded from shortening analyses. A platinum electrode was used to stimulate the selected fibres from 10 to 100 Hz, and we showed the maximum shortening velocity and minimum shortening length were basically the same in *Actn3KO* fibres and WT. During a sustained tetanic contraction, there will be a build-up of K^+^ ions in the restricted t-tubular extracellular space which can result in the failure of the t-tubular action potential [33]. In the present study, it is possible that if there was a failure of action potential transmission in the t-tubules it would cause us to underestimate the maximum shortening length. The results from the high-speed camera support our earlier findings which reported the *Actn3* polymorphism and resulting absence of α-actinin-3 did not alter the expression of the fast myosin isoform [12, 13, 18]; however, this is the first time this has been shown directly for unloaded shortening of fibres rather than inferred from the myosin type. For the skinned fibre experiments, we used the EDL muscle to get individual fibres which were long enough to manually tie to a force transducer biomechatronics system. Given ~ 79% of EDL fibres are of 2B MHC isoform, ~ 16% type 2X and ~ 4% type 2A [28], we have previously shown [12] that 2B fibres are around twice the diameter of the 2X and 2A fibres. Thus, by selecting the top 30% of the largest diameter fibres, we were confident in having 2B fibres. This was confirmed by the pCa-force curves which were consistent with fast-twitch type 2B fibre types (Fig. 2c). The similarity of the pCa-force curves between genotypes supports earlier findings that there are no changes in myosin isoforms [1]. α-Actinin proteins are a key component of the Z-discs anchoring actin fibres from adjacent sarcomeres and transmitting force longitudinally to the tendons [14, 30]; thus, the chemical skinned fibre technique, using whole chemically skinned fibres (as opposed to mechanically skinned fibre segments), is the ideal way to test the effect of absence of α-actinin-3 from the Z-discs in fast-twitch muscle. In this preparation, there is no interference from adjacent fibres, connective tissue or lateral transmission of force [30]. We measured resting length tension, stiffness and visco-elasticity using the *MyoRobot* biomechatronics skinned fibre set up [22]. We found no significant differences in these properties indicating that both α-actinin-2 and α-actinin-3 confer the same mechanical properties to Z-discs. In an elegant, skinned fibre study on *ACTN3KO* humans, Broos and Malisoux [34] showed in terms of their visco-elastic properties, fast-twitch 2X muscle fibres from α-actinin-3-positive humans were the same as those from humans who were α-actinin-3 negative. The group also showed that there was no difference in maximum specific force with respect to genotype, as we would predict from our earlier studies where we showed there was no change in myosin isoform expression with respect to genotype [1, 12, 13, 18]. Our current studies in the mouse confirm the human findings, and while we did see a trend towards less absolute force in *Actn3KO*, this was due to the fact, as confirmed by Broos and Malisoux [34], that *Actn3KO* fast fibres (2X humans, 2B mice) are significantly smaller in diameter, and when we normalised the force results for cross-sectional area, we found the maximal specific force was the same.

We have previously reported that when the isolated EDL muscles are subjected to five eccentric contractions of 20% + *L*_0_ strain, there is no effect of genotype on the eccentric contraction-induced force deficit [18]. Intriguingly, in a later study [19] when we used a stronger eccentric contraction protocol with a strain of 30% + *L*_0_, there was a significant increase in the eccentric contraction force deficit in the *Actn3KO* fast-twitch EDL muscles. In our current study, the permeabilised single fibre preparation excludes any effects of eccentric contractions on excitation–contraction coupling, or on sarcolemma and extracellular matrix structures that transmit force. This allows us to look the effect of the absence of alpha-actinin-3 from the Z lines has on the mechanical strength of the fibre without interference from these other elements normally present in the intact muscle. Direct comparisons between data from experiments on single permeabilised fibres and those on whole muscles are problematic due to the lower amount of eccentric stretch required in single fibres to induce comparable force deficits to intact muscle [35]. The 20% EC stretch used here is likely in the comparable range we have previously used on intact isolated EDL muscle preparations of 20–30%. Thus, the results from the current study provide a likely explanation for these whole muscle results as we show that in some cases fibres from both *Actn3KO* and WT EDL muscles can withstand three eccentric contractions of 20% strain; however, ~ 60% of the *Actn3KO* fibres broke during the procedure compared with ~ 35% of the WT fibres (Fig. 6b). *Second harmonic imaging* of single EDL fibres revealed a myofibrillar structural deformity which provides a plausible explanation for this increased mechanical instability associated with the *Actn3KO* genotype. *Actn3KO* fibres contained numerous axial lattice shift of adjacent myofibrils (verniers apparent as branched ‘Y’-patterns) of the myofibrillar contractile proteins, like the vernier deviations we have previously reported in unbranched dystrophic muscle fibres (termed “chaotic”) from the *mdx* mouse [23, 36]. Modelling from intact fibres [37] has shown that macroscopic branches within a fibre are points of mechanical weakness which may be susceptible to breakage when fibres were stressed by eccentric contractions. A recent publication [38] shows that the normal myofibrillar matric contains sarcomeres which cross link with each other by branching; interestingly, they report that postnatally this branching occurs more frequently in fast-twitch muscle fibres like the ones we are studying here. Our hypothesis is that once the amount of myofibrillar branching increases, as is the case in the Actn3KO fast-twitch fibres, the deviations caused by the myofibrillar branches themselves mechanically comprise the ability of the fibre to withstand the high forces sustained during eccentric contractions or large stretches [39, 40]. We propose that the *Actn3KO* fast fibres behave normally under moderate strains, but as the strain increases there comes a point where the weaker dislodged myofibril arrays start to snap, setting up a positive feedback loop placing additional stresses on the remaining VD which in turn break. This provides an explanation as to why the intact muscle was not damaged at 20% + *L*_0_ eccentric contraction strain [18], but showed a significant force loss at 30% + *L*_0_ [19], which in our model would be sufficient strain to rupture the weaker myofibrillar out-of-register lattice in the fast α-actinin-3-deficient fibres. We have previously shown the presence of internalised centralised nuclei at baseline in *Actn3KO* muscles. Centralised nuclei were not present in the age matched controls [19]. Centralised nuclei are an accepted histological marker of a regenerated fibre. This would suggest these fast-twitch fibres with increased myofibrillar lattice shifts are subject to damage during normal muscle contraction when compared to WT fast fibres containing α-actinin-3 in the Z-discs. There have been several reports in the literature that α-actinin-3-deficient individuals may experience faster decline in muscle function with increasing age [12, 41] and our results may explain some of this decline because as sarcopenia develops, the loss of muscle mass will place greater stress on the remaining fast-twitch muscles during eccentric contractions. In the case of individuals lacking α-actinin-3 protein, their remaining fast-twitch muscles will be at greater risk of damage compared to α-actinin-3 protein-positive individuals. This will be compounded by the reduced diameter of the fast-twitch muscles in the α-actinin-3 protein-deficient individuals, increasing mechanical axial stress on single fibres.

## Conclusion

1. Unloaded single fibre shortening length and maximum speed of shortening at different field-stimulation frequencies (10–100 Hz) are unaltered by the absence of α-actinin-3 in *Actn3KO* single intact muscle fibres from FDB fast-twitch muscle.
2. Using the chemically skinned fibre technique with single fast-twitch EDL fibres, we show visco-elastic properties and myofibrillar force production (force-pCa) are not affected by the absence of α-actinin-3.
3. When chemically skinned single EDL fibres were maximally activated and subjected to three eccentric contractions of Lo + 20% strain, ~ 60% of the *Actn3KO* fibres broke during the procedure compared with ~ 35% of the WT fibres.
4. Second harmonic imaging of single *Actn3KO* EDL fibres revealed myofibrillar structural abnormalities with an axial lattice shift of adjacent myofibrils (verniers apparent as ‘Y’-patterns).
5. The structural weakness caused by the Y-shaped vernier branches provides a plausible explanation for the increased mechanical instability associated with the *ACTN3* genotype.

## Supplementary Information

**Additional file 1: Supplementary Figure A. **Frequency distribution of FDB fibre widths. A total of 200 WT FDB fibres were measured using ImageJ.

**Additional file 2: Supplementary Figure B. **High-speed camera view of a single unloaded FDB fibre contracting at 20 Hz.

## Data Availability

The datasets used and/or analysed during the current study are available from the corresponding author on reasonable request.

## Abbreviations

CAS: Cosine angle sum
CSA: Cross-sectional area
dL: Distance shortened
dT: Time shortened
EDL: Extensor digitorum longus
*F*_abs_: Maximum absolute force
*F*_R_: Restoration force
*F*_relax_: Force at steady state
*F*_sig_: End of fast phase
FDB: Flexor digitorum brevis
*K*_apparent_: Apparent dissociation constant
KO: Knock out
*L*_0_: Optimal length
MyHC: Myosin heavy chain
pCa: Calcium concentration
pCa_50_: Calcium concentration at 50% of maximum force
SHG: Second harmonic generation
*τ*_relax_: Time to reach steady state
VD: Vernier density
*V*_fast_: Fast velocity
WT: Wild type

## References

[1] Berman Y, North KN (2010). A gene for speed: the emerging role of alpha-actinin-3 in muscle metabolism. Physiology (Bethesda).

[2] North KN, Yang N, Wattanasirichaigoon D, Mills M, Easteal S, Beggs AH (1999). A common nonsense mutation results in alpha-actinin-3 deficiency in the general population. Nat Genet.

[3] Head SI, Chan S, Houweling PJ, Quinlan KG, Murphy R, Wagner S (2015). Altered Ca2+ kinetics associated with α-Actinin-3 deficiency may explain positive selection for ACTN3 null allele in human evolution. PLoS Genet.

[4] Wyckelsma VL, Venckunas T, Houweling PJ, Schlittler M, Lauschke VM, Tiong CF (2021). Loss of alpha-actinin-3 during human evolution provides superior cold resilience and muscle heat generation. Am J Hum Genet.

[5] Knoll R (2002). Z-line proteins: implications for additional functions. Eur Heart Journal Supplements.

[6] Yang N, MacArthur DG, Gulbin JP, Hahn AG, Beggs AH, Easteal S (2003). ACTN3 genotype is associated with human elite athletic performance. Am J Hum Genet.

[7] Lee FXZ, Houweling PJ, North KN, Quinlan KGR (2016). How does α-actinin-3 deficiency alter muscle function? Mechanistic insights into ACTN3, the ‘gene for speed’. Biochim Biophys Acta.

[8] Friedrich O, Weber C, von Wegner F, Chamberlain JS, Fink RH (2008). Unloaded speed of shortening in voltage-clamped intact skeletal muscle fibers from wt, mdx, and transgenic minidystrophin mice using a novel high-speed acquisition system. Biophys J.

[9] Lannergren J, Westerblad H (1987). The temperature dependence of isometric contractions of single, intact fibres dissected from a mouse foot muscle. J Physiol.

[10] Lännergren J, Westerblad H (1991). Force decline due to fatigue and intracellular acidification in isolated fibres from mouse skeletal muscle. J Physiol.

[11] Stephenson DG, Williams DA (1982). Effects of sarcomere length on the force-pCa relation in fast- and slow-twitch skinned muscle fibres from the rat. J Physiol.

[12] Seto JT, Chan S, Turner N, MacArthur DG, Raftery JM, Berman YD (2011). The effect of α-actinin-3 deficiency on muscle aging. Exp Gerontol.

[13] Chan S, Seto JT, Houweling PJ, Yang N, North KN, Head SI (2011). Properties of extensor digitorum longus muscle and skinned fibers from adult and aged male and female Actn3 knockout mice. Muscle Nerve.

[14] Schiaffino S, Reggiani C (2011). Fiber types in mammalian skeletal muscles. Physiol Rev.

[15] Barbara V, De Katrien B, Monique R, Van den Els E, Van Marc L, Peter H (2007). ACTN3 (R577X) genotype is associated with fiber type distribution. Physiol Genomics.

[16] Lowe DA, Warren GL, Hayes DA, Farmer MA, Armstrong R (1994). Eccentric contraction-induced injury of mouse soleus muscle: effect of varying [Ca2+] o. J Appl Physiol.

[17] Warren GL, Hayes DA, Lowe DA, Armstrong RB (1993). Mechanical factors in the initiation of eccentric contraction-induced injury in rat soleus muscle. J Physiol.

[18] Chan S, Seto JT, MacArthur DG, Yang N, North KN, Head SI (2008). A gene for speed: contractile properties of isolated whole EDL muscle from an alpha-actinin-3 knockout mouse. Am J Physiol Cell Physiol.

[19] Seto JT, Lek M, Quinlan KG, Houweling PJ, Zheng XF, Garton F (2011). Deficiency of α-actinin-3 is associated with increased susceptibility to contraction-induced damage and skeletal muscle remodeling. Hum Mol Genet.

[20] MacArthur DG, Seto JT, Chan S, Quinlan KG, Raftery JM, Turner N (2008). An Actn3 knockout mouse provides mechanistic insights into the association between α-actinin-3 deficiency and human athletic performance. Hum Mol Genet.

[21] Hsu C, Moghadaszadeh B, Hartwig JH, Beggs AH (2018). Sarcomeric and non-muscle α-actinin isoforms exhibit differential dynamics at skeletal muscle Z-lines. Cytoskeleton (Hoboken).

[22] Haug M, Reischl B, Prolss G, Pollmann C, Buckert T, Keidel C (2018). The MyoRobot: a novel automated biomechatronics system to assess voltage/Ca(2+) biosensors and active/passive biomechanics in muscle and biomaterials. Biosens Bioelectron.

[23] Friedrich O, Both M, Weber C, Schurmann S, Teichmann MDH, von Wegner F (2010). Microarchitecture is severely compromised but motor protein function is preserved in dystrophic mdx skeletal muscle. Biophys J.

[24] Bekoff A, Betz WJ (1977). Physiological properties of dissociated muscle fibres obtained from innervated and denervated adult rat muscle. J Physiol.

[25] Head SI, Stephenson DG, Williams DA (1990). Properties of enzymatically isolated skeletal fibres from mice with muscular dystrophy. J Physiol.

[26] Selvin D, Hesse E, Renaud JM (2015). Properties of single FDB fibers following a collagenase digestion for studying contractility, fatigue, and pCa-sarcomere shortening relationship. Am J Physiol Regul Integr Comp Physiol.

[27] Yusuke Komiya1 SSDMRIMNRTYIWM (2017). Mouse soleus (slow) muscle shows greater intramyocellular lipid droplet accumulation than EDL (fast) muscle: fiber type-specific analysis. J Muscle Res Cell Motil.

[28] Hettige P, Tahir U, Nishikawa KC, Gage MJ (2020). Comparative analysis of the transcriptomes of EDL, psoas, and soleus muscles from mice. BMC Genomics.

[29] Williams DA, Head SI, Lynch GS, Stephenson D (1993). Contractile properties of skinned muscle fibres from young and adult normal and dystrophic (mdx) mice. J Physiol.

[30] Patel TJ, Lieber RL (1997). Force transmission in skeletal muscle: from actomyosin to external tendons. Exerc Sport Sci Rev.

[31] Nielsen J, Farup J, Rahbek SK, de Paoli FV, Vissing K (2015). Enhanced glycogen storage of a subcellular hot spot in human skeletal muscle during early recovery from eccentric contractions. PLoS One.

[32] Pyle WG, Solaro RJ (2004). At the crossroads of myocardial signaling: the role of Z-discs in intracellular signaling and cardiac function. Circ Res.

[33] Allen DG, Lamb GD, Westerblad H (2008). Skeletal muscle fatigue: cellular mechanisms. Physiol Rev.

[34] Broos S, Malisoux L, Theisen D, van Thienen R, Ramaekers M, Jamart C (2016). Evidence for ACTN3 as a Speed gene in isolated human muscle fibers. PloS One.

[35] Brooks SV, Faulkner JA (1996). The magnitude of the initial injury induced by stretches of maximally activated muscle fibres of mice and rats increases in old age. J Physiol.

[36] Buttgereit A, Weber C, Garbe CS, Friedrich O (2013). From chaos to split-ups - SHG microscopy reveals a specific remodelling mechanism in ageing dystrophic muscle. J Pathol.

[37] Iyer SR, Shah SB, Valencia AP, Schneider MF, Hernández-Ochoa EO, Stains JP (2017). Altered nuclear dynamics in MDX myofibers. J Appl Physiol.

[38] Willingham TB, Kim Y, Lindberg E, Bleck CKE, Glancy B (2020). The unified myofibrillar matrix for force generation in muscle. Nat Commun.

[39] Chan S, Head S (2011). The role of branched fibres in the pathogenesis of Duchenne muscular dystrophy. Exp Physiol.

[40] Diermeier S, Iberl J, Vetter K, Haug M, Pollmann C, Reischl B (2017). Early signs of architectural and biomechanical failure in isolated myofibers and immortalized myoblasts from desmin-mutant knock-in mice. Sci Rep.

[41] Judson RN, Wackerhage H, Hughes A, Mavroeidi A, Barr RJ, Macdonald HM (2011). The functional ACTN3 577X variant increases the risk of falling in older females: results from two large independent cohort studies. J Gerontol A Biol Sci Med Sci.

