# Supplementary figures and images for: Absence of the Z-disc protein α-actinin-3 impairs the mechanical stability of Actn3KO mouse fast-twitch muscle fibres without altering their contractile properties or twitch kinetics
Source: Skelet Muscle. 2022 Jun 23;12:14. doi: 10.1186/s13395-022-00295-8 (PMC9219180; doi:10.1186/s13395-022-00295-8)

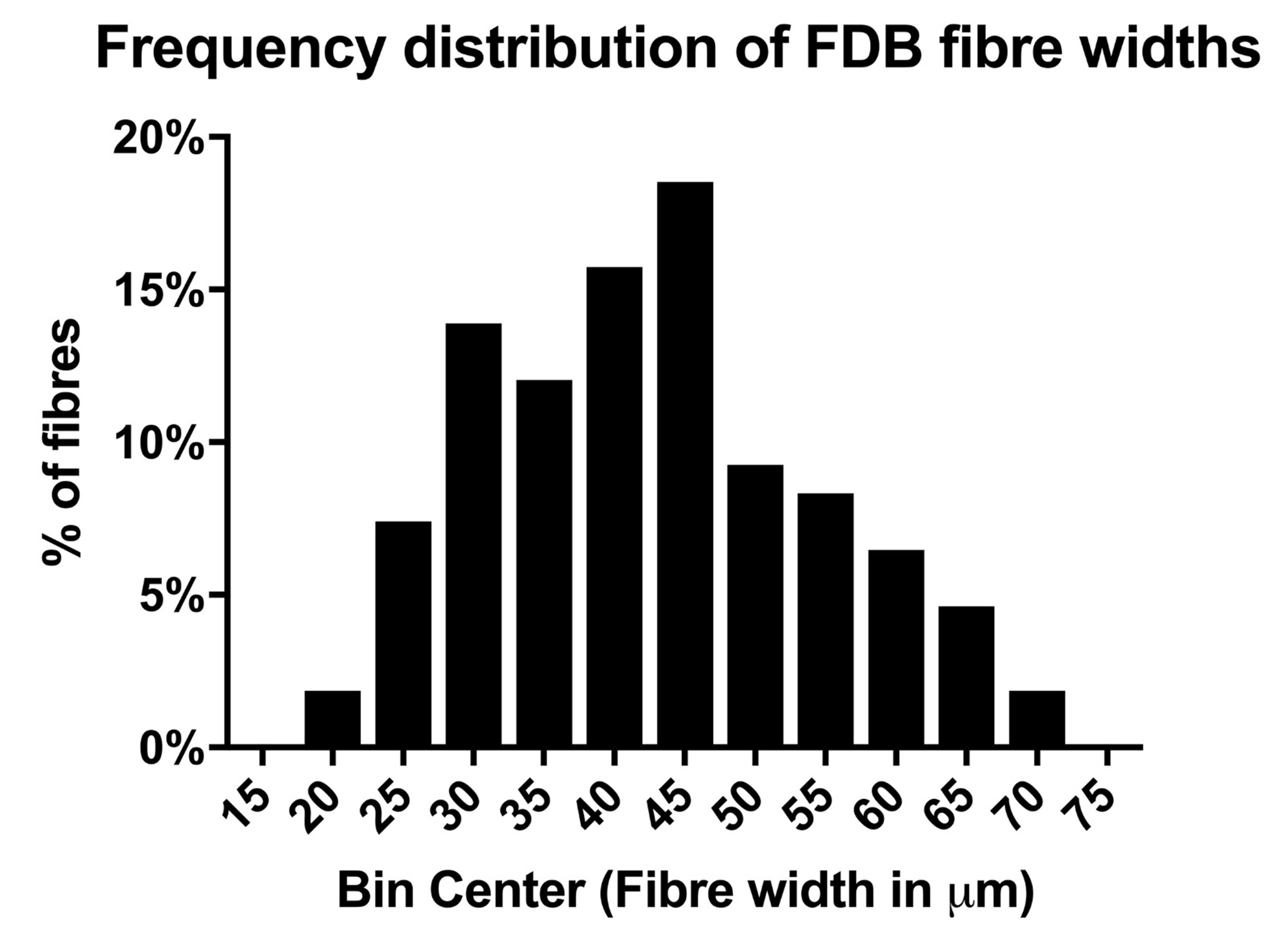

Supplement: Supplementary file 1 — Additional file 1: Supplementary Figure A. Frequency distribution of FDB fibre widths. A total of 200 WT FDB fibres were measured using ImageJ. [file 13395_2022_295_MOESM1_ESM.tif]
